# Author Correction: GATA3 and MDM2 are synthetic lethal in estrogen receptor-positive breast cancers

**DOI:** 10.1038/s42003-022-03612-5

**Published:** 2022-07-04

**Authors:** Gaia Bianco, Mairene Coto-Llerena, John Gallon, Venkatesh Kancherla, Stephanie Taha-Mehlitz, Mattia Marinucci, Martina Konantz, Sumana Srivatsa, Hesam Montazeri, Federica Panebianco, Vijaya G. Tirunagaru, Marta De Menna, Viola Paradiso, Caner Ercan, Ahmed Dahmani, Elodie Montaudon, Niko Beerenwinkel, Marianna Kruithof-de Julio, Luigi M. Terracciano, Claudia Lengerke, Rinath M. Jeselsohn, Robert C. Doebele, François-Clément Bidard, Elisabetta Marangoni, Charlotte K. Y. Ng, Salvatore Piscuoglio

**Affiliations:** 1 Visceral Surgery and Precision Medicine Research Laboratory, Department of Biomedicine, University of Basel, Basel, Switzerland; 2 Institute of Medical Genetics and Pathology, University Hospital Basel, Basel, Switzerland; 3 Department of Biomedicine, University of Basel and University Hospital Basel, Basel, Switzerland; 4 Department of Biosystems Science and Engineering, ETH Zurich, Basel, Switzerland; 5 Department of Bioinformatics, Institute of Biochemistry and Biophysics, University of Tehran, Tehran, Iran; 6 Rain Therapeutics Inc, Newark, CA USA; 7 Department of Biomedical Research, Urology Group, University of Bern, Bern, Switzerland; 8 Laboratory of Preclinical Investigation, Department of Translational Research, Institut Curie Research Center, Paris, France; 9 Department of Pathology, Humanitas Clinical and Research Center, IRCCS, Milan, Italy; 10 Department of Biomedical Sciences, Humanitas University, Milan, Italy; 11 Division of Women’s Cancers, Dana-Farber Cancer Institute, Harvard Medical School, Boston, MA USA; 12 Department of Medical Oncology, Institut Curie, Saint Cloud, France; 13 Department for BioMedical Research (DBMR), University of Bern, Bern, Switzerland; 14 SIB Swiss Institute of Bioinformatics, Lausanne, Switzerland

**Keywords:** Breast cancer, Breast cancer

Correction to: *Communications Biology* 10.1038/s42003-022-03296-x, published online 19 April 2022.

In this article the affiliation details for Marianna Kruithof-de Julio were incorrectly given as ‘Rain Therapeutics Inc, Newark, CA, USA’ but should have been ‘Department of Biomedical Research, Urology Group, University of Bern, Bern, Switzerland’. In this article the affiliation details for Elisabetta Marangoni were incorrectly given as ‘Department of Biomedical Research, Urology Group, University of Bern, Bern, Switzerland’ but should have been ‘Laboratory of Preclinical Investigation, Department of Translational Research, Institut Curie Research Center, Paris, France’. The original article has been corrected.

