# Supplementary material for: GATA3 and MDM2 are synthetic lethal in estrogen receptor-positive breast cancers
Source: Commun Biol. 2022 Apr 19;5:373. doi: 10.1038/s42003-022-03296-x (PMC9018745; doi:10.1038/s42003-022-03296-x)
Supplement: Supplementary file 2 — Supplementary Information [file 42003_2022_3296_MOESM2_ESM.pdf]

GATA3 and MDM2 are synthetic lethal in estrogen receptor-positive breast cancers

Gaia Bianco[1], Mairene Coto-Llerena[1,2], John Gallon[1], Venkatesh Kancherla[2], Stephanie Taha-Mehlitz[1], Mattia Marinucci[1], Martina Konantz[3], Sumana Srivatsa[4], Hesam Montazeri[2,5], Federica Panebianco[1], Vijaya G. Tirunagaru[6], Marta De Menna[7], Viola Paradiso[1,2], Caner Ercan[2], Ahmed Dahmani[8], Elodie Montaudon[8], Niko Beerenwinkel[4], Marianna Kruithof-de Julio[6], Luigi M. Terracciano[2,9,10], Claudia Lengerke[3], Rinath M. Jeselsohn[11], Robert C. Doebele[6], François-Clément Bidard[12], Elisabetta Marangoni[7], Charlotte K. Y. Ng[2,13,14*] and Salvatore Piscuoglio[1,2*]

Supplementary Information

Supplementary Figures 1-7

Supplementary Table 1

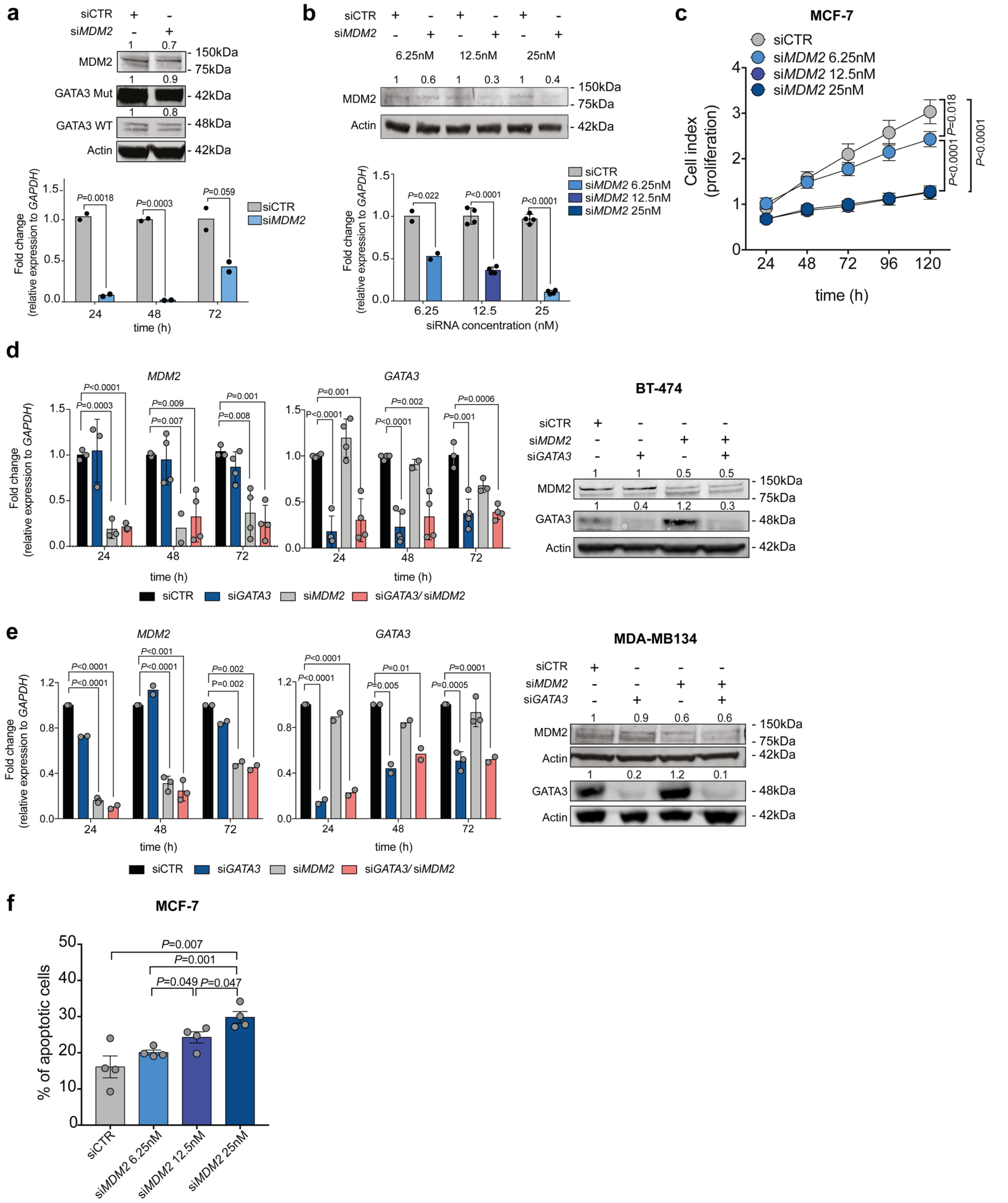

Supplementary Fig. 1: *GATA3* and *MDM2* are synthetic lethal in ER-positive breast cancer. (a) Immunoblot showing *GATA3*-mutant, *GATA3*-wild type and MDM2 protein expression levels in MCF-7 cells at 72 h post-siRNA transfection (upper panel). *MDM2* mRNA expression levels (relative to *GAPDH*) in MCF-7 cells at 24, 48 and 72 h post-siRNA transfection (bottom panel). **(b)** Immunoblot showing MDM2 protein expression levels at 72 h post-siRNA transfection (upper panel) and *MDM2* mRNA levels at 48 h post-siRNA transfection (bottom panel) after transfection with different concentrations of siRNA (6.25 nM, 12.5 nM or 25 nM) in MCF-7 cells. **(c)** Proliferation kinetics of MCF-7 cells transfected with *MDM2* siRNA at different concentrations. **(d,e)** *MDM2* and *GATA3* mRNA expression level (relative to *GAPDH*) in **(d)** BT-474 and **(e)** MDA-MB134 cells at 24, 48 and 72 h post-siRNA transfection (left panel). Immunoblot showing *MDM2* and *GATA3* protein levels of expression in **(d)** BT-474 and **(e)** MDA-MB134 cells 72 h post-siRNA transfection (right panel). **(f)**

Flow cytometry analysis of Annexin V and propidium iodide co-staining to measure the percentage of apoptotic cells (AnnV+) and live cells (AnnV-/PI-) upon *MDM2* silencing with different concentrations of siRNA in MCF-7 cells. For all the western blots, quantification is relative to the loading control (actin) and normalized to the corresponding siCTR control. Data are mean ± s.d. n≥2 biologically independent replicates. Statistical significance was determined for (**a,b,c,d,e**) by multiple t-test and for **(f)** by two-tailed unpaired Student's t-test.

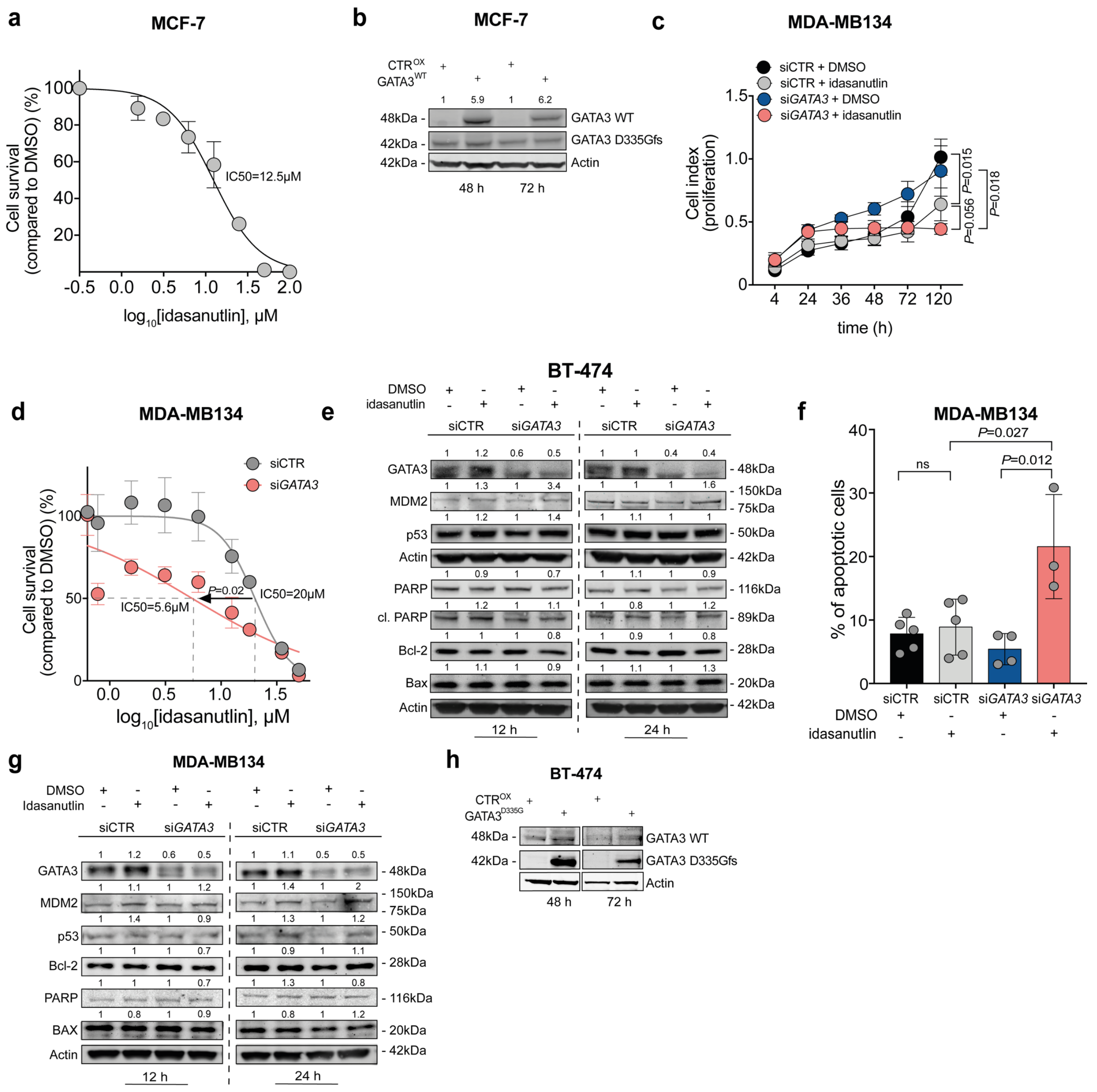

Supplementary Fig. 2: *GATA3* status determines response to MDM2 inhibitors *in vitro*. (a) Log-dose response curve of idasanutlin in MCF-7 cells. **(b)** Immunoblot of GATA3 in MCF-7 cells 48 and 72 hours post transfection with control or GATA_WT vector. (**c**) Effect of *GATA3* silencing on proliferation upon treatment with DMSO or idasanutlin 12.5 µM in MDA-MB134 cells. (**d**) Log-dose response curve of Idasanutlin in MDA-MB134 cells transfected with control siRNA or *GATA3* siRNA. (**e,g**) Immunoblot showing pro- and anti-apoptotic proteins at 12 and 24 h post-treatment with DMSO or Idasanutlin 12.5 µM in (**e**) BT-474 and (**g**) MDA-MB134 cells transfected with control siRNAs or *GATA3* siRNAs. **(f)** Percentage of apoptotic cells upon *GATA3* silencing and Idasanutlin treatment (12.5 µM) in MDA-MB134 cells. **(h)** Immunoblot of GATA3 in BT-474 cells 48 and 72 hours post transfection with control or GATA_D335Gfs vector. For all the western blots, quantification is relative to the loading control (actin) and normalized to the corresponding DMSO control. Data are mean ± s.d. n≥3 biologically independent replicates. Statistical significance was determined for (**c**) by multiple t-test and for **(d,f)** by the two-tailed unpaired Student's t-test.

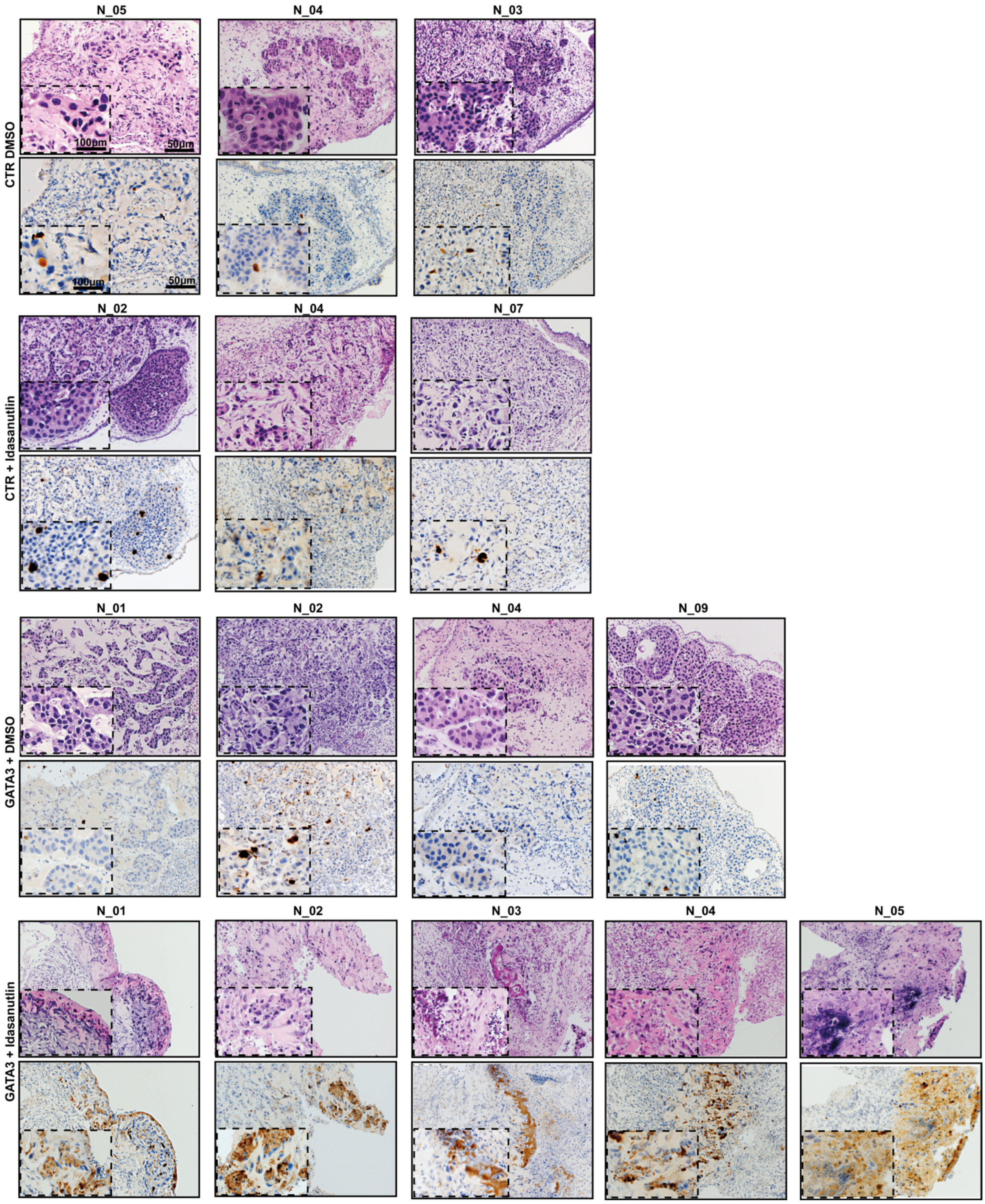

Supplementary Fig. 3: GATA3 expression determines response to MDM2 inhibitor *in vivo.* Representative micrographs of BT-474 tumors extracted four days post-implantation. Tumoural cells (hematoxylin/eosin; upper panel) were immunostained with the apoptotic marker cleaved caspase 3 (lower panel) in the different treatment conditions. Scale bars: 100 μm and 50 μm.

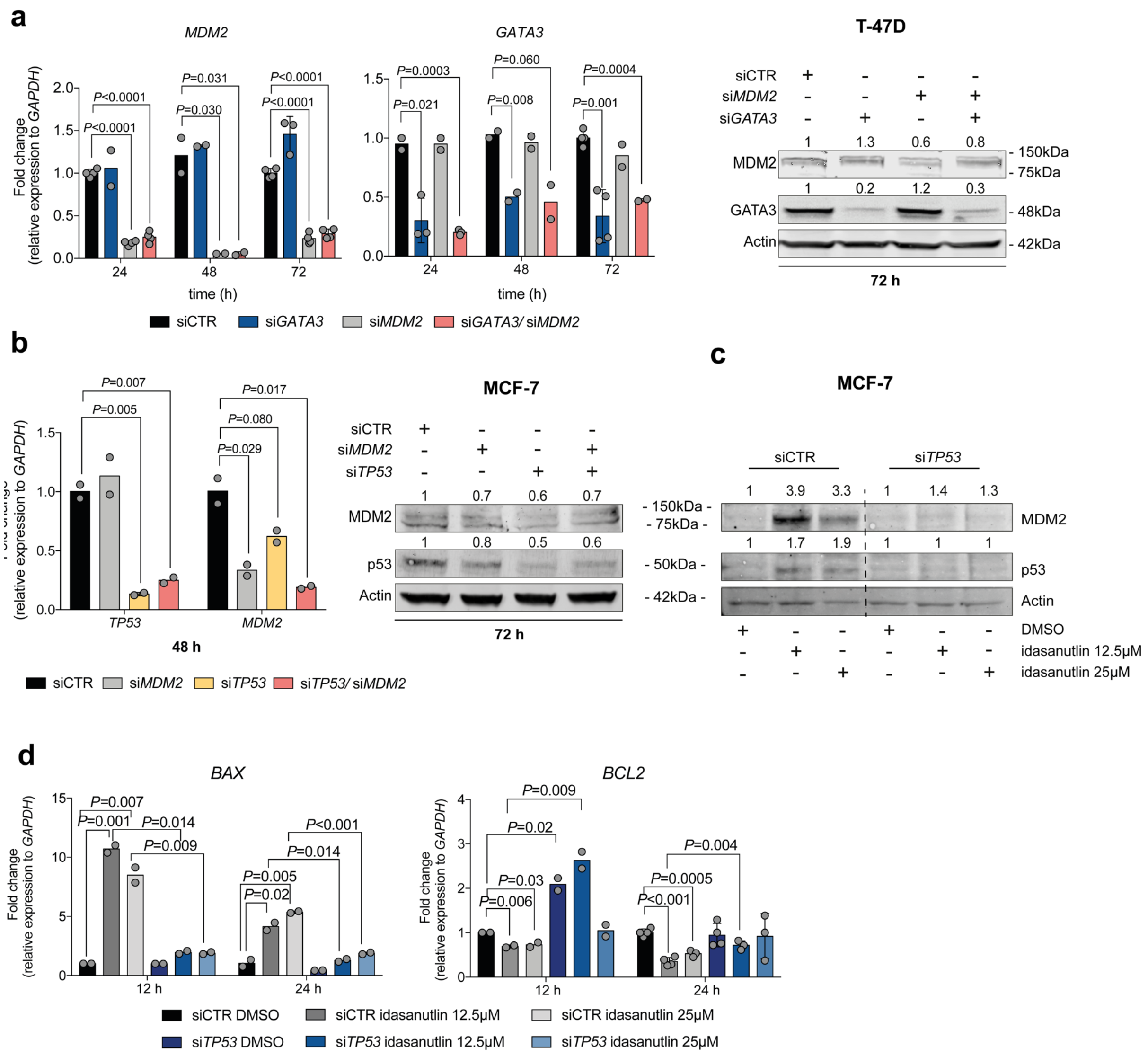

Supplementary Fig. 4: The synthetic lethality between *GATA3* and *MDM2* is *TP53* dependent. (a) *MDM2* and *GATA3* mRNA level of expression (relative expression to *GAPDH*) in T-47D at 24, 48 and 72 h post siRNA transfection (left panel). Immunoblot showing *MDM2* and *GATA3* protein level of expression in T-47D cells 72 h post-siRNA transfection (right panel). **(b)** *MDM2* and *TP53* mRNA level of expression (relative to expression of *GAPDH*) in MCF-7 cells 48 h post-siRNA transfection (left panel). Immunoblot showing MDM2 and p53 protein levels of expression in MCF-7 cells 72 h post-siRNA transfection (right panel). **(c)** Immunoblot showing MDM2 and p53 protein levels 24 h post-treatment with DMSO or idasanutlin (12.5 µM) in MCF-7 cells transfected with control siRNAs or *TP53* siRNAs. **(d)** mRNAs levels of *BCL2* and *BAX* in control and *TP53*-silenced MCF-7 cells at 12 and 24 h post-treatment. For all the western blots, quantification is relative to the loading control (actin) and normalized to the corresponding DMSO control. Data are mean ± s.d. n≥2 biologically independent replicates. Statistical significance was determined for **(a,b,d)** by multiple t-test.

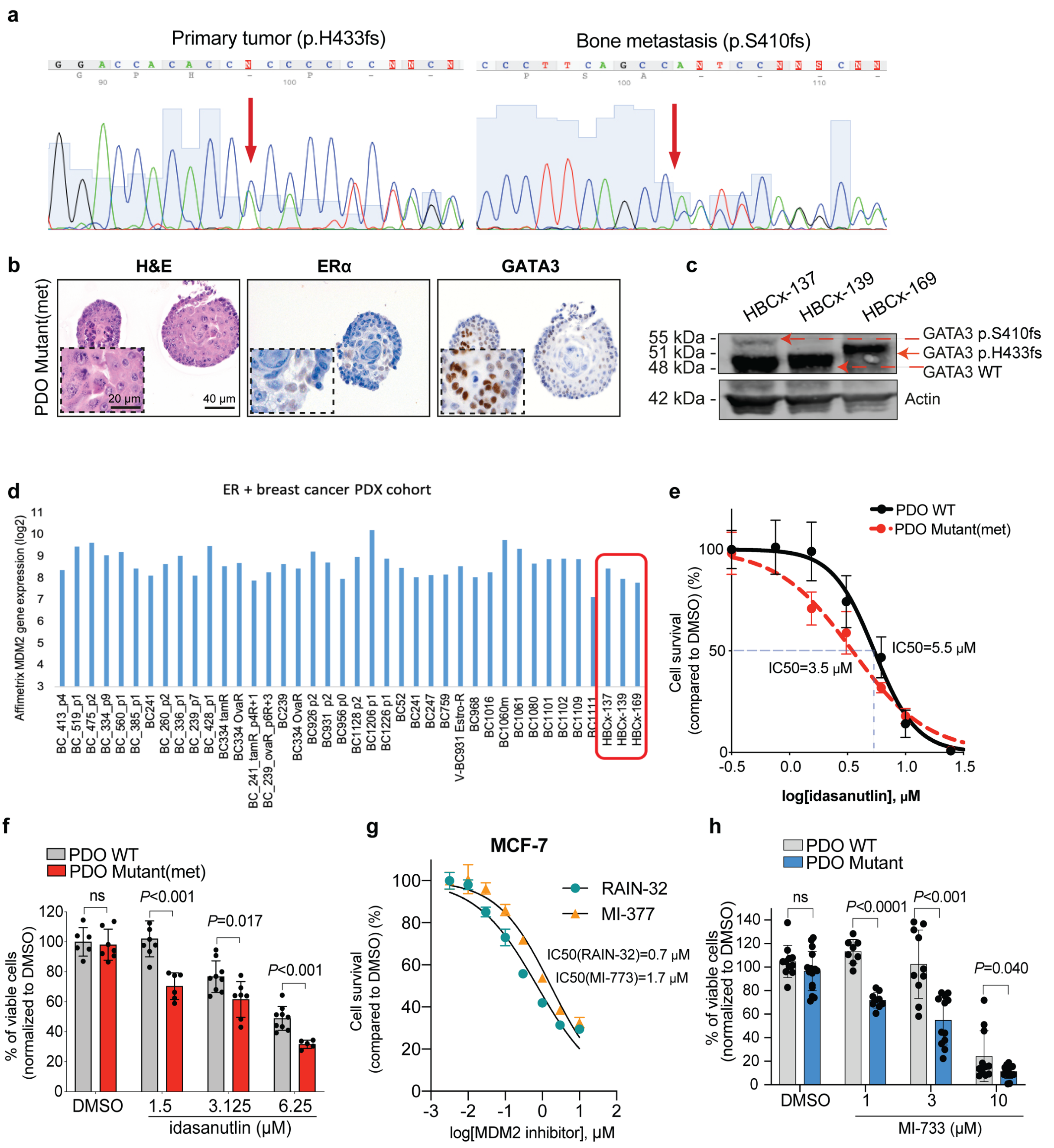

Supplementary Fig. 5: *GATA3* mutations predict response to MDM2 inhibitors in ER-positive breast cancer PDOs and PDX. (**a**) Chromatogram obtained from the Sanger sequencing of the *GATA3* gene in the primary (left) and metastatic (right) ER-positive *GATA3*-mutant breast cancer PDOs. (**b**) Representative micrographs of H&E, ERα and GATA3 immuno-staining on the *GATA3*-mutant (met) PDO. (**c**) Immunoblot of GATA3 in tissue derived from PDXs carrying wild-type GATA3 (HBCx-139), *GATA3* mutant p.S410fs (HBCx-137), and *GATA3* mutant p.H433fs (HBCx-169). (**d**) *MDM2* gene expression (log2, Affymetrix array) in the ER-positive breast cancer PDX cohort of the Institut Curie. (**e**) Log-dose response curve of idasanutlin in *GATA3* wild-type (IC50=5.4 μM) or *GATA3*-mutant (met) (3.5 μM) PDOs. (**f**) Percentage of viable cells upon treatment with different dosages of idasanutlin in *GATA3* wild-type or *GATA3*-mutant (met) PDOs. (**g**) Log-dose response curve of MI-733 and RAIN-32 in MCF-7 cells. (**h**) Percentage of viable cells upon treatment with different dosages of MI-733 in *GATA3* wild-type or *GATA3*-mutant (met) PDOs. Scale bars are 20 and 40 μm for (**b**). Data are mean ± SD, n≥4 biologically independent replicates. Statistical significance was determined for **(e,f,h)** by multiple t-test.

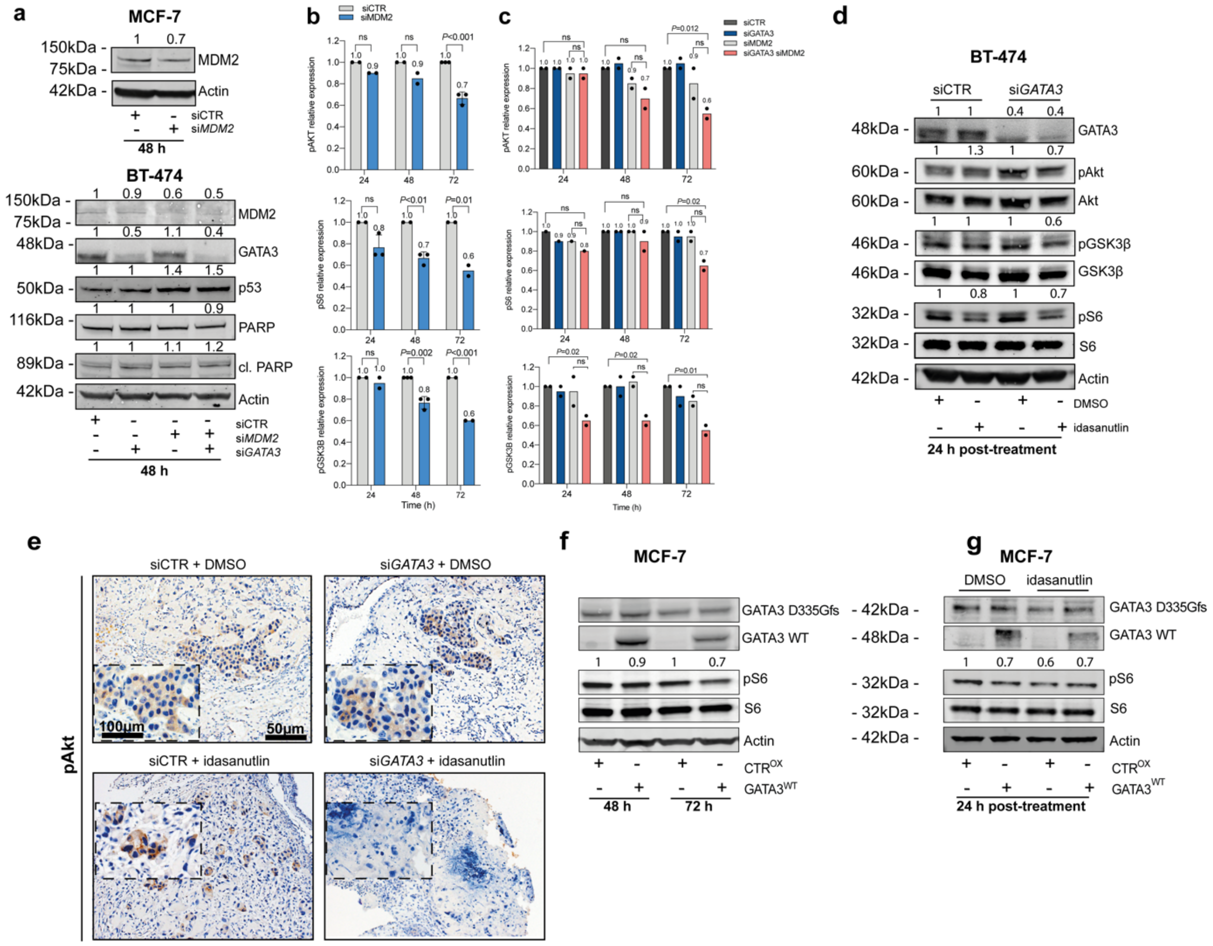

Supplementary Fig. 6: The synthetic lethality between *GATA3* and *MDM2* acts via the PI3K-Akt-mTOR signaling pathway. (a) Immunoblot showing MDM2 protein expression in MCF-7 (upper) and MDM2, GATA3, p53, PARP and cleaved PARP protein expression in BT-474 cells (bottom). (**b-c**) Statistical analysis (two-tailed unpaired Student's t-test) of p-AKT, p-GSK3B and p-S6 relative expression from the immunoblots in Fig. 6c-d. **(d)** Immunoblot showing markers of mTOR signaling pathway activation at 48 h post siRNA transfection and 24 h post-treatment with DMSO or Idasanutlin in BT-474 cells. Note that this immunoblot was performed using the same membrane as the immunoblot for 24 h in Supplementary Fig. 2c. (**e**) Representative immunohistochemistry of phospho-Akt staining in BT-474 tumors extracted four days post-implantation. Scale bars: 100 µm and 50 µm. (**f**) Immunoblot showing mutant (D335Gfs) and wild-type GATA3, actin, total and phospho-S6 (mTOR signaling pathway activation) at 48 and 72h post control or GATA3 wild-type overexpression in MCF-7 cells. (**g**) Immunoblot showing mutant (D335Gfs) and wild-type GATA3, actin, total and phospho-S6 (mTOR signaling pathway activation) at 48 h post control or GATA3 wild-type overexpression, and 24 h post treatment with vehicle or idasanutlin (12.5 µM) in MCF-7 cells. For all the western blots, quantification is relative to the loading control (actin) and normalized to the corresponding silencing/overexpression control or DMSO control.

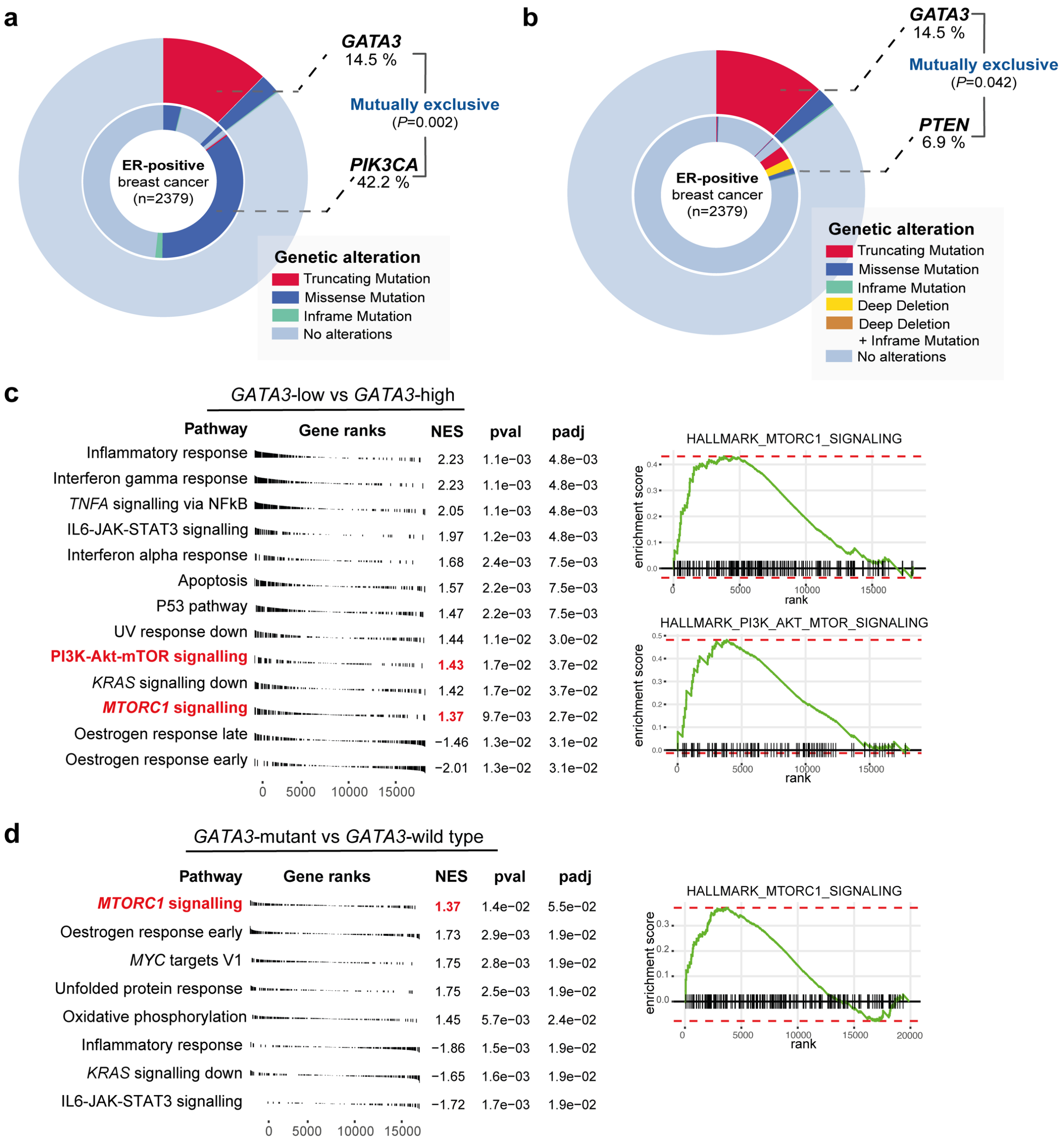

Supplementary Fig. 7: *GATA3*, *PIK3CA* and *PTEN* molecular status in ER positive breast cancers. Doughnut charts showing the mutual exclusivity between (**a**) *GATA3* and *PIK3CA* and (**b**) *GATA3* and *PTEN* genetic alterations in ER-positive breast cancer patients. Mutational data for the *GATA3, PIK3CA* and *PTEN* genes and copy number status for *PTEN* were derived from the TCGA PanCancer Atlas [1] and the METABRIC datasets [4]. (**c,d**) Normalized enrichment scores of significantly up- and down-regulated pathways identified by gene set enrichment analysis **(c)** in ER-positive breast cancers with low *GATA3* versus high *GATA3* expression and **(d)** in ER-positive breast cancers with *GATA3*-mutant versus *GATA3* wild-type. Statistical significance was determined for **(a,b)** by one-sided Fisher's Exact test and for **(c,d)** by *fgsea*[89].

Supplementary Table 1: Clinical characteristics and immunophenotypic features of the BR5496 tumor sample.

Model name	Cancer Type	Human Race	RNAseq	Generation
BR5496	Breast	Caucasian	P0/WE,NA	P8

Model name	Subtype	*TP53* mutation	*GATA3* mutation	Ethnicity	Pathology Diagnosis	Pathology QC
BR5496	ER(+), PR(+)	None	p.D335Gfs	Western	Breast carcinoma	Invasive ductal carcinoma, with necrosis(P0)
