# Supplementary material for: GATA3 and MDM2 are synthetic lethal in estrogen receptor-positive breast cancers
Source: Commun Biol. 2022 Apr 19;5:373. doi: 10.1038/s42003-022-03296-x (PMC9018745; doi:10.1038/s42003-022-03296-x)
Supplement: Supplementary file 3 — Description of Additional Supplementary Files [file 42003_2022_3296_MOESM3_ESM.pdf]

Description of Additional Supplementary Files

File name: Supplementary Data 1
Description: List of 22 breast cancer cell lines included in the DRIVE analysis and their mutations in GATA3, PIK3CA, PIK3R1, PTEN, FOXA1, ESR1 and ARID1A.

File name: Supplementary Data 2
Description: Table of SLIdR results and effect sizes for all possible synthetic lethal (SL) partners of GATA3 from Project DRIVE. For each possible SL partner, SLIdR uses one-sided statistical tests based on the Irwin-Hall distribution to test whether the viabilities of GATA3 mutated cell lines from knockdown of the SL partner gene are lower than expected by chance. “Mean viability of GATA3-mutant cell lines” and the “Mean viability of GATA3-wild-type cell lines” are the Page 7 of 20 average viabilities from the knockdown of each possible SL partner in GATA3 mutated and WT cell lines, respectively. The effect size is under the “Difference in viabilities” column. The resulting p-values are reported in column “p value” with the false discovery rate shown under “FDR”.

File name: Supplementary Data 3
Description: List of all the reagents (including antibodies, chemicals, peptides, recombinant proteins, commercial assays, oligonucleotides and qPCR primers) used in this study.

File name: Supplementary Data 4
Description: Source data for graphs, charts and western blots.
