# Supplementary material for: GATA3 and MDM2 are synthetic lethal in estrogen receptor-positive breast cancers
Source: Commun Biol. 2022 Apr 19;5:373. doi: 10.1038/s42003-022-03296-x (PMC9018745; doi:10.1038/s42003-022-03296-x)
Supplement: Supplementary file 8 — Reporting Summary [file 42003_2022_3296_MOESM8_ESM.pdf]

nature research

Corresponding author(s): Salvatore Piscuoglio
Charlotte KY Ng

Last updated by author(s): Dec 23, 2021

Reporting Summary

Nature Research wishes to improve the reproducibility of the work that we publish. This form provides structure for consistency and transparency in reporting. For further information on Nature Research policies, see Authors & Referees and the Editorial Policy Checklist.

Statistics

For all statistical analyses, confirm that the following items are present in the figure legend, table legend, main text, or Methods section.

n/a	Confirmed	
☐	☒	The exact sample size (*n*) for each experimental group/condition, given as a discrete number and unit of measurement
☐	☒	A statement on whether measurements were taken from distinct samples or whether the same sample was measured repeatedly
☐	☒	The statistical test(s) used AND whether they are one- or two-sided<br>*Only common tests should be described solely by name; describe more complex techniques in the Methods section.*
☒	☐	A description of all covariates tested
☒	☐	A description of any assumptions or corrections, such as tests of normality and adjustment for multiple comparisons
☐	☒	A full description of the statistical parameters including central tendency (e.g. means) or other basic estimates (e.g. regression coefficient) AND variation (e.g. standard deviation) or associated estimates of uncertainty (e.g. confidence intervals)
☒	☐	For null hypothesis testing, the test statistic (e.g. *F*, *t*, *r*) with confidence intervals, effect sizes, degrees of freedom and *P* value noted<br>*Give P values as exact values whenever suitable.*
☒	☐	For Bayesian analysis, information on the choice of priors and Markov chain Monte Carlo settings
☒	☐	For hierarchical and complex designs, identification of the appropriate level for tests and full reporting of outcomes
☒	☐	Estimates of effect sizes (e.g. Cohen's *d*, Pearson's *r*), indicating how they were calculated

Our web collection on statistics for biologists contains articles on many of the points above.

Software and code

Policy information about availability of computer code

Data collection	N/A
Data analysis	Flow cytometry data were analysed by FlowJo software version 10.5.3.<br>Statistical analyses were conducted using Prism software v7.0 and R version 3.6.1.

For manuscripts utilizing custom algorithms or software that are central to the research but not yet described in published literature, software must be made available to editors/reviewers. We strongly encourage code deposition in a community repository (e.g. GitHub). See the Nature Research guidelines for submitting code & software for further information.

Data

Policy information about availability of data

All manuscripts must include a data availability statement. This statement should provide the following information, where applicable:

- Accession codes, unique identifiers, or web links for publicly available datasets
- A list of figures that have associated raw data
- A description of any restrictions on data availability

RNA-sequencing data are available at https://www.ncbi.nlm.nih.gov/sra/ (PRJNA623723).

Field-specific reporting

Please select the one below that is the best fit for your research. If you are not sure, read the appropriate sections before making your selection.

☒ Life sciences ☐ Behavioural & social sciences ☐ Ecological, evolutionary & environmental sciences

For a reference copy of the document with all sections, see nature.com/documents/nr-reporting-summary-flat.pdf

Life sciences study design

All studies must disclose on these points even when the disclosure is negative.

Sample size	For the in vivo experiments the samples size was calculated using a G*Power calculation.<br><br>For zebrafish experiments, assuming a difference of 20% in tumourigenic potential and type I error of 5%, 85 samples in each group would ensure >80% power to detect statistical differences between experimental groups using Fisher's exact test. Furthermore, assuming a 95% engraftment rate, 95 experiments would ensure we had >95% probability of having 85 successful xenotransplantations.<br><br>For the CAM assay, assuming an effect size of 1.5 and type I error of 5%, 9 samples in each group would ensure >80% power to detect statistical differences between experimental groups using unpaired t-tests. Furthermore, assuming a 95% engraftment rate, 10 experiments would ensure we had >91% probability of having 9 successful xenotransplantations.
Data exclusions	For the in vivo experiments, samples were excluded when the organism was in bad conditions (e.g. infected egg, undeveloped embryos) or when cells injection failed for technical reasons (e.g. for the Zebrafish experiment, fish were screened shortly post-injection for the presence of fluorescence at the site of injection).
Replication	All the experiments were performed at least three times and reported in the manuscript only when reproducible.
Randomization	For the in vivo experiments, fish and CAMs were allocated randomly to each condition.
Blinding	For the Zebrafish in vivo model, fish were screened for tumour formation blindly by two independent scientists. All the immunohistochemical stains were evaluated blindly by two independent pathologists.

Reporting for specific materials, systems and methods

We require information from authors about some types of materials, experimental systems and methods used in many studies. Here, indicate whether each material, system or method listed is relevant to your study. If you are not sure if a list item applies to your research, read the appropriate section before selecting a response.

Materials & experimental systems

n/a	Involved in the study	
☐	☒	Antibodies
☐	☒	Eukaryotic cell lines
☒	☐	Palaeontology
☐	☒	Animals and other organisms
☒	☐	Human research participants
☒	☐	Clinical data

Methods

n/a	Involved in the study	
☒	☐	ChIP-seq
☐	☒	Flow cytometry
☒	☐	MRI-based neuroimaging

Antibodies

Antibodies used	Primary antibodies: GATA3 (clone EPR16651)-abcam ref#ab199428 (working dilution for WB 1 : 1000; IHC 1:500); MDM2 (clone 2A10) MerkMillipore ref#MABE281 (WB 1 : 50); p53 (DO-1) abcam ref# ab1101 (WB 1 : 250); BCL-2 Cell signalling (CST) ref#2872S (WB 1 : 1000); PARP/cl.PARP Cell signalling (CST) ref#9542 (WB 1 : 1000); BAX (D2E11) Cell signalling (CST) ref# 5023 (WB 1 : 1000); Akt Cell signalling (CST) ref#9272S (WB 1 : 1000); phospho-AKT (Ser473) (clone D9E) Cell signalling (CST) ref#4060S (WB 1 : 1000; IHC 1:100); S6 ribosomal protein (clone 5G10) Cell signalling (CST) ref# 2217S (WB 1 : 1000); phospho-S6 ribosomal protein (Ser235/236) (clone D57.2.2E) Cell signalling (CST) ref#4858S (WB 1 : 2000; IHC 1:400); GSK-3beta (clone D5C5Z) Cell signalling (CST) ref# 12456S (WB 1 : 1000); phospho-GSK-3beta (Ser21/9) Cell signalling (CST) ref# 9331S (WB 1 : 1000); Actin (clone AC-15) Sigma ref# A5441 (WB 1 : 2000).<br>Secondary antibodies: goat anti-mouse (IRDye 680) LI-COR Biosciences; goat anti-rabbit (IRDye 800) LI-COR Biosciences.
Validation	All the primary antibodies were previously validated by commercial providers and previously described in literature. In detail:

GATA3 (ab199428) validated for ChIP, WB, IHC-P, ICC/IF, Flow Cyt (Brown S et al. Nature 548:334-337 (2017). WB ; Mouse .; MDM2 (MABE281) validate for WB and IP (Khosravi, R., et al. (1999). PNAS. 96(26):14973–14977); p53 (ab1101) was KO validated and tested for ChIP, ICC/IF, ELISA, IHC-P, IHC-Fr, IP, WB, Flow Cyt (Teng Z et al. Oncol Lett 17:1559-1564 (2019); BCL-2 (2872S) validate for WB (Dammert MA et al. Nat Commun. 2019 Aug 2;10(1):3485); PARP/cl.PARP (9542) validate for WB (Li N et al. Nucleic Acids Res. 2020 Apr 6;48(6):3014-3028); BAX (5023) validate for WB, IP, IHC (Zhang H et al. Oncol Lett. 2019 Dec;18(6):6261-6268); GSK-3beta (12456S) validate for WB, IHC-P, ICC/IF, Flow Cyt (Fan FY et al. Oncol Lett. 2019 Dec;18(6):6339-6346); phospho-GSK-3beta (9331S) validate for WB, IHC-P, ICC/IF, Flow Cyt (Cui D et al. Cell Death Differ. 2020 Mar;27(3):1119-1133); Akt (9272S), phospho-Akt (4060S), S6 (2217S) and phospho-S6 (4858S) were used as previously described in Panebianco et al. Proc Natl Acad Sci U S A. 2017 Feb 28;114(9):2307-2312); Actin (A5441) validate for WB (Senthil K Radhakrishnan et. al Oncogene, 23(23) (2004-3-30).

Eukaryotic cell lines

Policy information about cell lines

Cell line source(s)

MCF-7, BT-474, MDA-MB134 and T-47D were kindly provided by Dr. Rachael Natrajan from The Institute of Cancer Research (London, UK). MCF-7 cell lines with knock-in mutations in the ESR1 gene (p.Y537S and p.D538G) were provided by Dr. Jeselsohn (eselsohn, R. et al. Mutations. Cancer Cell 33, 173–186.e5 (2018).

Authentication

Cells were authenticated by short tandem repeat profiling.

Mycoplasma contamination

Cell lines were monitored regularly for mycoplasma contamination by PCR using specific primers as described previously (Uphoff, C. C. & Drexler, H. G. Methods Mol. Biol. 731, 93–103 (2011).

Commonly misidentified lines
(See ICLAC register)

N/A

Animals and other organisms

Policy information about studies involving animals; ARRIVE guidelines recommended for reporting animal research

Laboratory animals

Zebrafish, fertilized chicken eggs and NOD/SCID mice were used in this study.
Zebrafish wild-type Tuebingen strains were used. Zebrafish were bred and maintained as described previously (Nusslein-Volhard, C. & Dahm, R. (Oxford University Press, 2002)). Staging was done by hours post-fertilization (hpf) as described previously (Kimmel, C. B., Warga, R. M. & Schilling, T. F. Development 108, 581–594 (1990)), and according to FELASA and Swiss federal law guidelines.
Fertilized chicken eggs were obtained at day 1 of gestation and were maintained at 37°C in a humidified (60%) incubator for 13 days.

PDX tumor fragments of 2 to 3 mm in diameter were subcutaneously xenografted into NOD/SCID mice. Each mouse was inoculated subcutaneously at the right flank region with BR5496 tumor chunk for tumor development. 40 mice were enrolled in the study. The PDX-inoculated mice were selected and randomly categorized into vehicle, fulvestrant, RAIN-32 50 mg/kg or RAIN-32 100 mg/kg groups (eight mice per group) when the mean tumor size reached approximately 144 mm3. The treatments started when the mean tumor size reached approximately 150 mm3 and lasted for 29 days. Randomization was performed based on the “Matched distribution” method using the StudyDirectorTM software, version 3.1.399.19 randomized block design.

Wild animals

N/A

Field-collected samples

N/A

Ethics oversight

Animal experiments and zebrafish husbandry were approved by the “Kantonales Veterinaeramt Basel-Stadt” (haltenewilligung: 1024H) in Switzerland.
Fertilized chicken eggs were obtained at day 1 of gestation and were maintained for 13 days. Therefore, according to Swiss federal laws, no ethical approval is required.

All mouse experiments were approved by and performed in accordance with the guidelines and regulations of the Animal Ethics Committee of the Association for Assessment and Accreditation of Laboratory Animal Care International. NOD/SCID female mice of 6-8 weeks of age were housed under pathogen-free conditions in individually ventilated cage (IVC) systems at constant temperature and humidity at the animal facilities of Crown Bioscience, Inc.

Note that full information on the approval of the study protocol must also be provided in the manuscript.

Flow Cytometry

Plots

Confirm that:

☒ The axis labels state the marker and fluorochrome used (e.g. CD4-FITC).

☒ The axis scales are clearly visible. Include numbers along axes only for bottom left plot of group (a 'group' is an analysis of identical markers).

☒ All plots are contour plots with outliers or pseudocolor plots.

☒ A numerical value for number of cells or percentage (with statistics) is provided.

Methodology

Sample preparation	Breast cancer cells were harvested after incubation period and washed twice by centrifugation (1,200g, 5mins) in cold phosphate-buffered saline. After washing, cells were resuspended in 0.1ml AnnV binding buffer 1X containing fluorochrome-conjugated AnnV and PI (PI to a final concentration of 1μg/ml) and incubated in darkness at room temperature for 15mins. As soon as possible cells were analysed by flow cytometry, measuring the fluorescence emission at 530nm and >575nm.
Instrument	BD FACSCanto II cytometer (BD Biosciences, USA)
Software	Data were analysed by FlowJo software version 10.5.3.
Cell population abundance	N/A
Gating strategy	Cells were first gated using the preliminary FSC-A/SSC-A axes. Douplets were excluded gating FSC-A/FSC-W axes. Finally cells were gated using Annexin V/PI axes defining live cells (bottom left quadrant; AnnV -/PI -); necrotic cells (upper left quadrant; AnnV -/PI+); early apoptotic (bottom right panel; Annex V +/PI -); late apoptotic (upper right quadrant; AnnV +; PI +). For the total number number of apoptotic cells (%) both early and late apoptotic cells were counted.

☒ Tick this box to confirm that a figure exemplifying the gating strategy is provided in the Supplementary Information.
